# Magnesium, Little Known But Possibly Relevant: A Link between NASH and Related Comorbidities

**DOI:** 10.3390/biomedicines9020125

**Published:** 2021-01-27

**Authors:** Jorge Simón, Teresa Cardoso Delgado, Luis Alfonso Martinez-Cruz, Maria Luz Martínez-Chantar

**Affiliations:** 1 Liver Disease Laboratory, Center for Cooperative Research in Biosciences (CIC bioGUNE), Basque Research and Technology Alliance (BRTA), Bizkaia Technology Park, Building 801A, 48160 Derio, Bizkaia, Spain; tcardoso@cicbiogune.es (T.C.D.); amartinez@cicbiogune.es (L.A.M.-C.); 2 Centro de Investigación Biomédica en Red de Enfermedades Hepáticas y Digestivas (CIBERehd), 48160 Derio, Bizkaia, Spain

**Keywords:** non-alcoholic steatohepatitis (NASH), magnesium (Mg^2+^), obesity, insulin resistance (IR), type 2 diabetes mellitus (T2DM), hypertension, cardiovascular diseases (CVD)

## Abstract

Non-alcoholic steatohepatitis (NASH) is characterized by an abnormal hepatic lipid accumulation accompanied by a necro-inflammatory process and a fibrotic response. It comprises from 10% to 30% of cases of patients with non-alcoholic liver disease, which is a global health problem affecting around a quarter of the worldwide population. Nevertheless, the development of NASH is often surrounded by a pathological context with other comorbidities, such as cardiovascular diseases, obesity, insulin resistance or type 2 diabetes mellitus. Dietary imbalances are increasingly recognized as the root cause of these NASH-related comorbidities. In this context, a growing concern exists about whether magnesium consumption in the general population is sufficient. Hypomagnesemia is a hallmark of the aforementioned NASH comorbidities, and deficiencies in magnesium are also widely related to the triggering of complications that aggravate NASH or derived pathologies. Moreover, the supplementation of this cation has proved to reduce mortality from hepatic complications. In the present review, the role of magnesium in NASH and related comorbidities has been characterized, unraveling the relevance of maintaining the homeostasis of this cation for the correct functioning of the organism.

## 1. Introduction

### 1.1. Non-Alcoholic Steatohepatitis: An Overview

Non-alcoholic steatohepatitis, or NASH, is a term used to define a pathophysiological stage of the liver characterized by an abnormal lipid accumulation (steatosis), inflammation, hepatocellular damage and fibrosis development [1]. NASH is included in the group of conditions that define the spectrum of non-alcoholic fatty liver disease (NAFLD), together with non-alcoholic fatty liver (NAFL or steatosis) and cirrhosis [2]. NAFLD has an estimated prevalence of 25% in the worldwide population, whereas NASH is estimated to affect 3–12% of the global population [3]. The highest rates of NAFLD are reported in South America and the Middle East, followed by Asia, USA and Europe, and they are expected to increase within the next years due to current lifestyle and dietary habits [3,4]. Remarkably, the development of NAFLD/NASH has been widely characterized as a risk factor for the development of hepatocellular carcinoma (HCC), contributing to 10–12% of cases in Western populations, and 1–6% of cases in Asian populations [5,6]. HCC is the second leading cause of cancer-related death and the fifth most common type of cancer worldwide [7]. Furthermore, both NASH and NAFLD patients usually present an elevated risk not only of liver-related morbidity and mortality, but also other metabolic comorbidities, such as insulin resistance (IR) and type 2 diabetes mellitus (T2DM), hypertension and cardiovascular diseases (CVD) or obesity [3].

The liver plays a key role in the metabolism of all biomolecules, but perturbations in lipid balance lead to the development of steatosis. In NAFLD, the genetic background or nutritional imbalances lead to a downregulation of the pathways involved in hepatic lipid clearance: (i) very-low-density lipoprotein (VLDL) secretion and (ii) fatty acid oxidation (FAO) or an upregulation in those that promote hepatic lipid content as (iii) de novo lipogenesis (DNL) and (iv) fatty acid (FA) uptake [8]. Although steatosis is usually considered a “brand” condition, chronic abnormal lipid deposition together with other hepatic insults or even other events beyond the liver, that include, for example, gut dysbiosis and adipose tissue inflammation, contribute to the development of NASH. In this context, the most common lipid-derived complications comprise of the excessive development of reactive oxygen species (ROS) and oxidative stress, the appearance of endoplasmic reticulum stress (ERS), mitochondrial dysfunction with subsequent decreased FAO capacity, and the production of lipotoxic species, which are of relevance [2,9]. These hits promote the development of liver fibrosis, characterized by an excessive extracellular matrix (ECM) deposition because of a chronic damage and a wound healing response [10]. In such an environment, hepatocytes suffer from an inflammatory and apoptotic signaling that leads to their death [11], activating Kupffer cells (KCs) to promote the release of inflammatory cytokines [12] and hepatic stellate cells (HSCs) to secrete ECM components [13].

### 1.2. Nutritional Imbalances in Non-Alcoholic Steatohepatitis: A Potential Role of Magnesium

Unhealthy nutritional habits and dietary imbalances are beginning to be recognized as the root cause of many diseases. Particularly, it was previously mentioned that NASH has metabolic perturbations that are the most common cause of development [14]. High-fat and high-sugar diets, such as the Western diet, lead to an increased fatty acid uptake by the liver and adipose tissue while promoting hepatic DNL [15]. Although an excessive calorie intake leads to the spread of overweight and obesity worldwide, currently affecting 38% of the worldwide population and expected to increase in the coming years [16], unhealthy dietary habits are also often accompanied by some imbalances in certain nutrients. In particular, deficiencies in dietary micronutrients have been identified.

Magnesium, or Mg^2+^ in its free form, is a micronutrient widely distributed in the food supply, both in plant and animal foods. Most green vegetables, legumes, peas, beans and nuts are rich in magnesium, as are some shellfish and spices. The daily recommended intake (DRI) for elemental magnesium is age-dependent, and lower for women (e.g., 19–30 years: 310 mg; 31 years and older: 320 mg) than men (e.g., 19–30 years: 400 mg; 31 years and older: 420 mg). Magnesium DRI increases for pregnant women and lactation periods. The cation is absorbed in the duodenum and ileum by both active and passive processes, and this process is affected by different nutrients, such as fiber content [17]. Under healthy conditions, the kidney plays a central role in magnesium homeostasis. In the last few years, a growing concern has emerged about the defective magnesium consumption in the general population, as, according to the National Health and Nutrition Examination Survey (NHANES), 79% of US adults do not meet the DRI of the cation [18].

Mg^2+^ is the most predominant divalent cation in the cell, with concentrations ranging from 5–20 mM, and extracellular Mg^2+^ accounts for only 1% of the total content in the organism [19,20]. Magnesium plays a role as cofactor in more than 300 enzymatic reactions, especially in those involving adenosine-triphosphate (ATP) or guanosine triphosphate (GTP), where the cation forms ATP-Mg or GTP-Mg stable complexes required for many biological processes such as glucose stabilization, lipogenesis, protein synthesis, nucleic acids synthesis, coenzymes activity or methylation, among others [19]. The maintenance of magnesium homeostasis is crucial for the correct development of the organism, whereas perturbations have been related to the triggering of an inflammatory response, mitochondrial dysfunction and the decrease of the antioxidant capacity [21]. These alterations in such biological processes have been reported to occur in comorbidities associated with NASH such as obesity, hypertension, CVD and/or the development of IR or T2DM. Indeed, perturbations in Mg^2+^ homeostasis have been reported, not only in liver pathologies but also in concomitant systemic complications [22,23,24].

In the present review we aim to highlight the relevance of Mg^2+^ homeostasis and the development of systemic complications accompanying NASH. 

## 2. Magnesium and Systemic Complications during Non-Alcoholic Steatohepatitis

### 2.1. Magnesium in Non-Alcoholic Fatty Liver Disease and Cancer

The spread of unhealthy lifestyle habits, together with inadequate nutritional behavior, are making NAFLD and the pathologies comprised in its spectrum the leading cause of chronic liver disease worldwide. Although, to date, the role of Mg^2+^ in the development of NASH has not been explored in depth, a clinical study showed a protective effect of Mg^2+^ intake in patients with liver diseases. Remarkably, an increased intake of 100 mg (25–33% increase of the daily recommended intake) showed a 49% reduction of liver-derived mortality [25], suggesting a possible role of the cation depletion in the development of liver diseases. Related to cirrhosis, the most severe stage of the pathologies that comprise the spectrum of NAFLD, decreased hepatic magnesium levels have been reported to promote collagen deposition in the liver, a hallmark of liver fibrosis [26,27]. The role of the cation is of relevance in the mitochondria from hepatocytes, where low intramitochondrial magnesium content has been described in cirrhosis with the subsequent decreased ATP production and increased hepatocellular damage [28]. The role of protein kinase Cε (PKCε) and its relationship with Mg^2+^ has also been characterized, as hypomagnesemia leads to a deficient PKCε translocation and subsequent fibrinogen and collagen deposition [29]. There is an existing link between low intrahepatic magnesium levels and an increased inflammatory response, as under low hepatic Mg^2+^ content, an over-activation of leukocytes and macrophages has been characterized, together with the recruitment of more inflammatory cells to the liver [30]. Moreover, the supplementation of this cation has been proposed as anti-cirrhotic therapy, because in vivo studies have demonstrated its protective effect [31].

As aforementioned, the development of NASH and other pathologies ranging in NAFLD may increase the risk of developing HCC. Related to this, the biological functions of the cation have been characterized, as Mg^2+^ plays a key role in DNA synthesis. Therefore, hypomagnesemia leads to dysfunctions in DNA damage repair mechanisms, modulation of cell cycle progression, cell proliferation and differentiation, and apoptosis with the subsequent tumor growth promotion and metastasis of the tumor [32,33]. Although molecular mechanisms that link hypomagnesemia and tumor development have not been elucidated yet, an in vitro study suggests a possible relationship between magnesium levels and a dysregulation of mitogen-activated protein kinase (MAPK)/extracellular signal-related kinase (ERK) signaling [34].

Finally, a continuous magnesium loss has been related to alcohol consumption [35], leading to the aggravation of alcoholic liver disease [26]. Related to this, Mg^2+^-deficient livers show an increased KC activation through the toll-like receptor type 4 (TLR4), promoting ROS generation [36] and pro-inflammatory cytokine release [37]. Remarkably, Mg^2+^ supplementation in patients with alcoholic steatohepatitis prevents the progression of the disease, reducing the transaminases levels in serum and decreasing liver-related morbidity [38].

**Figure 1:**
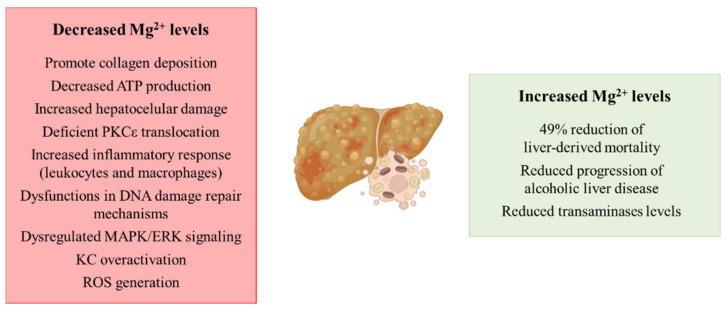
Schematic representation of the contribution of decreased and increased magnesium levels to the development of liver diseases. Abbreviations: PKCε: protein kinase Cε; MAPK: mitogen-activated protein kinase; ERK: extracellular signal-related kinase; KC: Kupffer cell; ROS: reactive oxygen species.

In summary, even though several studies suggest the relationship between hypomagnesemia and the development of liver diseases (Figure 1), to date there is no evidence that characterizes the relationship between the cation and NASH, so it could be a research topic of interest.

### 2.2. Magnesium in Overweight and Obesity

Overweight and obesity are complex and multifactorial diseases that affect over 1/3 of the world population. Similar to the epidemiology of NASH and NAFLD, these conditions are expected to even increase to a 38% prevalence of overweight and a 20% prevalence of obesity by 2030 [39]. Obesity is defined by an excessive body weight, in consequence of an excessive adiposity or body fatness. Healthy individuals show a body mass index (BMI) comprised between 18.5 and 24.99, whereas the BMI of overweight patients is between 25 and 29.99. Regarding the obese population, they can be classified into three subgroups: class I (BMI from 30 to 34.99), class II (BMI from 35 to 39.99) and class III (BMI higher than 40) [39]. Obesity greatly increases the risk of developing other chronic diseases such as those mentioned in the present manuscript: liver diseases (NASH/NAFLD), IR, T2DM, hypertension and CVD. Indeed, the development of NASH and overweight/obesity seem to follow a parallel direction. The risk of developing metabolic-derived liver complications has been reported to be two-fold higher in obese patients when compared with healthy non-obese patients [40].

Related to the possible role that Mg^2+^ perturbations may have in the development of alterations in obesity, hypomagnesemia has been identified in serum from obese patients [41]. Remarkably, a higher consumption of the cation is associated with a lower BMI, waist circumference and serum glucose levels in patients [42]. Related to the effect of the supplementation of the magnesium cation, it has been reported to improve the metabolic profile of normal-weight metabolically obese individuals in several parameters such as blood pressure (BP), fasting glucose and serum triglyceride levels [43]. Other studies have associated low magnesium concentrations with chronic inflammatory stress related to obese subjects, where hypomagnesemia enhances tumor necrosis factor (TNF) and interleukin-6 (IL-6) expression that might even contribute to the aggravation of other pathologies such as hepatic ones [44]. Additionally, Mg^2+^ consumption prevents the induction of obesity in in vivo models, suggesting a pivotal role of the cation in maintaining energy homeostasis [45]. However, other studies highlight the relevance of accompanying magnesium supplementation with a protein-sparing modified low-calorie diet in order to avoid the loss of the cation from the organism, as the supplementation alone cannot obtain this goal [46]. Nevertheless, the supplementation of the cation has been deeply characterized to improve the metabolic profile of obese individuals by decreasing the amount of low-density lipoprotein (LDL) and increasing high-density lipoprotein (HDL) levels [47].

Finally, other studies have focused on the impact of magnesium modulation in the adipocyte. It has been known for a long time that the adipocyte is not only a simple storage depot for body energy, but in fact an endocrine organ playing a very relevant role in obesity [48]. Interestingly, magnesium supplementation has been shown to result in a shift by increasing the frequency distribution of large adipocytes whereas magnesium deficiency did not modify adipocyte size but increased their number in in vivo models of obesity [49]. This is far more relevant considering that the age-related changes in adipose tissue lipid storage may lead to the proliferation of adipocytes in order to sustain lipid accumulation, making more difficult the loss of adipose tissue in elder patients [49]. In fact, the adipose tissue expansion by adipocyte proliferation has been characterized to be feasible for promoting obesity development [50] and magnesium levels appear to modulate this adipocyte proliferation. 

**Figure 2:**
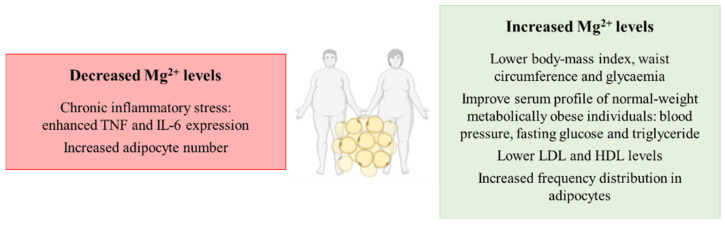
Schematic representation of the contribution of decreased and increased magnesium levels to the development of overweight or obesity. Abbreviations: TNF: tumor necrosis factor; IL-6: interleukin-6; LDL: low-density lipoprotein; HDL: high-density lipoprotein.

Overall, hypomagnesemia can contribute to the development of obesity, whereas supplementation with the Mg^2+^ cation appears to prevent and reduce obesity prevalence by targeting inflammation and adipocyte proliferation (Figure 2).

### 2.3. Magnesium in Insulin Resistance and Type 2 Diabetes Mellitus 

Insulin Resistance (IR) and type 2 Diabetes mellitus (T2DM) constitute together with obesity the main worldwide global epidemics, termed “diabesity”. Whereas the prevalence rates of IR syndrome have been reported to be 3–16%, with a higher incidence in white populations, the global prevalence of T2DM has been estimated to be 6.3% of the world’s population, being higher in elder patients [51]. IR is primarily an acquired condition related to an excessive body fat, being characterized by a deficient response to insulin leading to the reduction of glucose incorporation in the liver, adipose tissue and muscle. Therefore, glucose levels in serum increase and the pancreas synthesizes more insulin in order to overcome this perturbation. IR precedes the development of T2DM by 10–15 years of chronicity, in the case of a persistent hyperglycemia and an impaired insulin secretion [52]. The elevated levels of endogenous insulin are associated with IR and results in weight gain, which exacerbates IR in turn, fueling a vicious cycle that persists, with pancreatic β-cells that cannot meet the insulin demand. Furthermore, the deficient glucose incorporation by the three aforementioned tissues (adipose tissue, liver and muscle) has an impact in promoting hepatic gluconeogenesis through the impaired insulin secretion [52]. Likewise, this promotes DNL, contributing to steatosis development and blunting hepatic homeostasis.

Hypomagnesemia in both IR and T2DM patients has been described, as they often show not only a reduced intake of the cation but they also present an augmented urinary loss [22,53]. As mentioned earlier, urinary excretion of magnesium by the kidneys is very pertinent, and both hyperglycemia and hyperinsulinemia in T2DM increase urinary excretion and decrease tubular Mg^2+^ reabsorption [54]. T2DM patients in particular have also been characterized to present alterations in the status of the cation, especially in poorly controlled glycemic patients [55]. For instance, it has been estimated that around 50% of T2DM patients suffer from hypomagnesemia [56]. Related to this, more elevated glycated hemoglobin levels, as a marker of chronic T2DM development, are frequently observed in those elder patients that are more prone to suffer from hypomagnesemia [23]. The development of hepatic nephropathy as a T2DM-derived complication is also promoted under hypomagnesemia conditions [57]. Related to the molecular mechanisms that connect magnesium and the development of IR/T2DM, the cation plays a role as a co-factor in the phosphatidylinositol-3-kinase/protein kinase B (PI3K/AKT) pathway by which insulin exerts its role in peripheral tissues [58]. The transition from a transient IR stage to T2DM development might imply hypomagnesemia as a potential mediator, as Mg^2+^ deficiencies have been reported to alter the pancreatic insulin recreation by disrupting the normal activity of β-cells [59]. In β-cells, the insulin secretion is mainly controlled by the GLUT2/glucokinase (GK) tandem that acts as a glucose sensor and Mg^2+^ directly affects the GK activity [56]. Thus, hypomagnesemia impacts the ATP-sensitive K^+^ (KATP) channels that depolarize the β-cell membrane for the insulin release, leading to an uncontrolled insulin secretion [59]. Furthermore, hypomagnesemia may also reduce insulin sensitivity by promoting oxidative stress and/or inflammation as free radicals are often increased in T2DM [60] and pro-inflammatory cytokines such as IL-6 decrease the expression of GLUT4 and the activity of the PI3K pathway responsible in insulin-mediated signaling [61]. Remarkably, the effect of Mg^2+^ perturbations over insulin resistance development has been reported to be reversible, so that the recovery of normal levels of the cation could solve the pathology [62]. In this context, Mg^2+^ supplementation shows a significant improvement in plasma glucose, lipoprotein and triglycerides profile and BP in diabetic patients [63].

**Figure 3:**
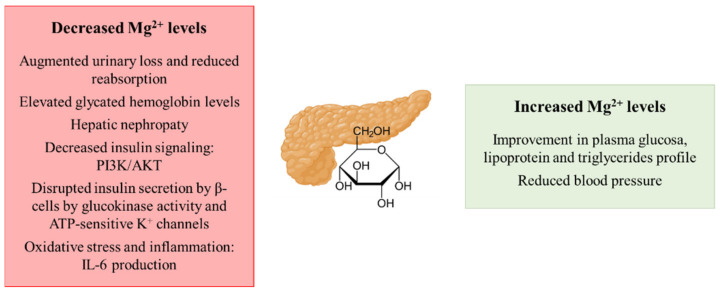
Schematic representation of the contribution of decreased and increased magnesium levels to the development of insulin resistance and type 2 diabetes mellitus. Abbreviations: PI3K: phosphatidylinositol-3-kinase; AKT: protein kinase B.

Therefore, as summarized in Figure 3, a depletion of magnesium, favored by the reduced intake as well as its continuous excretion by the urinary system together with a decreased reabsorption, may contribute to IR and T2DM development. On the contrary, the supplementation of the cation has been shown to exert a protective effect both on IR and T2DM.

### 2.4. Magnesium in Hypertension and Cardiovascular Diseases (CVD)

Hypertension is a growing health concern that over a decade ago was already estimated to affect around 31.1% of the global population (1.39 billion patients) [64]. This condition is characterized by an elevated BP and can be caused by many risk factors, such as high sodium intake, low potassium intake, obesity, alcoholic consumption, physical inactivity and an unhealthy diet [64]. Hypertension can be classified into stage 1 and stage 2 hypertension if systolic pressure is between 130–140, or higher than 140, respectively. Interestingly, elevated glucose levels caused either by IR or T2DM are also a risk factor of developing this condition [65]. Apart from being a major public health problem, hypertension is a major risk factor for the development of CVDs as it promotes left ventricular hypertrophy [66]. CVDs are by far the leading cause of death in the world, with 17.9 million deaths in 2015, which is expected to be at 22.2 million deaths by 2030 [67]. Concerning the different alterations that comprise the spectrum of CVDs, the most relevant are coronary artery disease, cerebrovascular disease, peripheral artery disease and aortic atherosclerosis [68]. Similar to hypertension, unhealthy lifestyle habits such as smoking, alcohol consumption and dietary imbalances highly increase the risk of developing CVDs [69]. Moreover, cardiovascular mortality is the most important cause of metabolic syndrome-related deaths.

Regarding the involvement of magnesium perturbations in the development of hypertension and cardiovascular diseases, there are several works than point out a relationship between the two conditions. Similar to the other conditions previously mentioned, the supplementation of the cation reduces BP in case of IR, pre-T2DM and other non-communicable chronic diseases [70]. Remarkably, a higher effect is observed in those patients with the most elevated BP [71]. Additionally, Mg^2+^ deficiencies are related to a reduction in endothelial functionality, a risk factor for atherosclerosis [24,72], while the supplementation regulates vascular tone and calcification, preventing atherogenesis and thrombosis development [73]. The cation is essential for many physiological, biochemical and cellular processes that regulate cardiovascular function, modulating vascular smooth muscle tone and the endothelial function [24]. Investigators also have characterized that Mg^2+^ plays a role in the proliferation and migration of endothelial and vascular muscle cells, the modulation of neuronal excitation and intracardiac conduction, as well as the myocardial contraction [73]. During cardiac potential, Mg^2+^ plays a role in the depolarization–repolarization processes, in that Mg^2+^ depletion alters electrocardiogram activity [74]. Low serum levels of the cation are related to increased atherosclerosis, coronary artery disease, arrhythmias and heart failure [73]. Correlated to this, lower intracellular levels of Mg^2+^ in skeletal muscle are related to aortic distensibility [75] and the appearance of arrhythmias [76]. The cation has been also reported to be essential for maintaining cell membrane potential, mitochondrial integrity from cardiomyocytes and to play a role in anti-oxidative pathways. [77]. Patients with T2DM that show perturbations in Mg^2+^ levels show alterations in echocardiographic indices and increased heart volume [78]. Similar to other studies in obese, IR or T2DM patients, the supplementation of Mg^2+^ in patients with CVD has also been reported to exert a protective role [77]. Houston and colleagues characterized that Mg^2+^ acts as a calcium channel blocker, increasing the production of nitric oxide (NO), improving endothelial dysfunction and inducing vasodilatation [79]. Additionally, scientific evidence highlights the relationship between Mg^2+^ deficiencies and an elevated risk of stroke [24]. Regarding the mechanisms that contribute to the aforementioned processes related to hypomagnesemia, the development of oxidative and inflammatory stress is of relevance [22], together with the contribution of the lipid profile and the increased susceptibility of lipoproteins to peroxidation [80]

**Figure 4:**
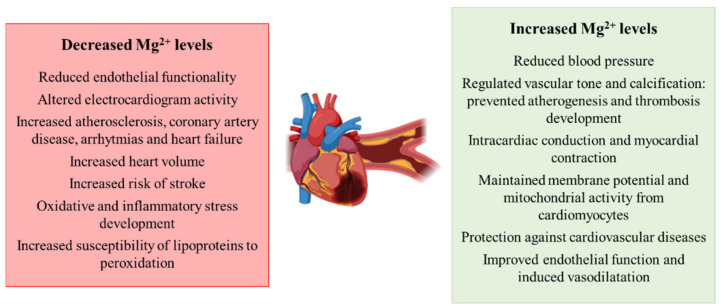
Schematic representation of the contribution of decreased and increased magnesium levels to the development of hypertension and cardiovascular diseases.

Overall, magnesium appears to play a role in the development of CVDs as deficiencies in the cation are related to atherogenesis and perturbations in skeletal muscle functionality (Figure 4).

## 3. Discussion

Between the systemic complications that normally accompany NASH development, obesity, IR and TD2M, hypertension and CVDs are the most common and relevant [3]. Indeed, it is evident that a relationship exists among all of them, as the development of one pathology is often accompanied by another. The development of NASH is frequently the consequence of a chronic overweight or obese state, whereas the metabolic disorders’ consequence of obesity lead to dysregulations that disturb glucose homeostasis, leading to IR or T2DM. In the meantime, elevated glucose levels in serum are also another cause of hypertension, where dysregulations in lipid metabolism lead to atherogenesis and promote the development of CVDs. In such an environment, the existence of an oxidative and inflammatory stress condition contributes to the development or aggravation of all aforementioned pathologies. Related to this, the role of Mg^2+^ deficiencies has been widely characterized.

Although NASH and related pathologies have their own specific complications, studies about the role of magnesium point in the same direction. Low magnesium concentrations have been related to many liver pathologies as cirrhosis, liver cancer or alcoholic liver disease. Moreover, hypomagnesemia is related to the development of a higher BMI—characteristic of obesity development—a higher risk of developing T2DM, and the development of atherogenesis and alterations in the cardiac muscle that lead to CVDs. Indeed, in the case of the pancreas, the cation has been characterized to play a role in insulin secretion by β-cells so that the loss of Mg^2+^ could contribute to the transition from IR to T2DM [59] and, in the meantime, decrease the insulin response from peripheral tissues [61]. Cardiomyocytes and endothelial cells in the cardiovascular system also require the cation for their correct functioning [77,79]. Another common feature of the cation in all of the conditions is the fact that the supplementation of the subjects, either in vivo or in clinical samples, prevents the development of such conditions. It must be mentioned that the role of the cation in NASH development has not been totally elucidated to date. However, the supplementation of the cation reduces the risk of liver-related death [25] so a positive effect is expected. Similarly, such supplementation also exerts a protective role over obesity, IR or T2DM and CVD development. Herein, the aforementioned role of Mg^2+^ in the prevention of oxidative and inflammatory stress could be a common mechanism. Moreover, considering the impact of mitochondrial dysfunction in the NASH-related comorbidities, other potential underlying mechanisms include the role of magnesium in the regulation of mitochondrial function. Finally, as the Mg^2+^ content can influence Ca^2+^ levels, and, for example, it has been shown that T2DM may cause endothelial dysfunction by remodeling the intracellular Ca^2+^ toolkit [81], this mechanism should be taken into account when investigating the protective roles of Mg^2+^ in IR, T2DM, CVD, and other NASH-related comorbidities.

On the one hand, considering the preventive role of the supplementation of Mg^2+^, the encouragement of the global population by health authorities to increase their nutritional intake of Mg^2+^ may reduce the prevalence of the pathologies mentioned in the present work. This can be achieved either by supplementation or by promoting the consumption of magnesium-rich food, such as green vegetables, nuts, seeds and unprocessed cereals [82]. Considering the information from NHANES that reveals that 79% of the adult population does not fulfill the DRI of 300–400 mg/day, this is a key point that preventive actions should focus on [18]. The potential effectivity of magnesium supplementation may be a research topic of interest towards the development of therapies. Thus, it would be interesting to evaluate its properties in either preclinical or clinical studies. 

On the other hand, the physicochemical properties of the cation make essential the activity of Mg^2+^ transporters that allow its flux across cell membranes participating in the processes of intake, excretion and reabsorption [83] In this aspect, although several transporters such as the cyclin M family (CNNM), magnesium transporter 1 (MagT1), MRS2 or the solute carrier family 41 (SLC41) have been characterized [84,85,86], their potential role in the development of NASH and related comorbidities remains largely unknown. CNNMs have been associated with a number of genetic diseases affecting ion flux and cancer development via their association with phosphatases of regenerating liver (PRL) [87], whereas deficiencies in MagT1 have been linked to immunodeficiencies [88]. Otherwise, mutations in MRS2 have been linked to demyelination [89] and SLC41 are related to abnormal locomotor functioning [90]. Related to the role of perturbations of Mg^2+^ homeostasis in the development of several diseases in which immune responses or essential biological processes are altered, the role of these proteins might be a research topic of interest for therapy development against NASH or related morbidities.

## Data Availability

Not Applicable.

## References

[1] Marra F., Lotersztajn S. (2013). Pathophysiology of NASH: Perspectives for a targeted treatment. Curr. Pharm. Des..

[2] Simon J., Ouro A., Ala-Ibanibo L., Presa N., Delgado T.C., Martínez-Chantar M.L. (2020). Sphingolipids in non-alcoholic fatty liver disease and hepatocellular carcinoma: Ceramide turnover. Int. J. Mol. Sci..

[3] Younossi Z., Anstee Q.M., Marietti M., Hardy T., Henry L., Eslam M., George J., Bugianesi E. (2018). Global burden of NAFLD and NASH: Trends, predictions, risk factors and prevention. Nat. Rev. Gastroenterol. Hepatol..

[4] Mishra A., Younossi Z.M. (2012). Epidemiology and Natural History of Non-alcoholic Fatty Liver Disease. J. Clin. Exp. Hepatol..

[5] Bertot L.C., Adams L.A. (2019). Trends in hepatocellular carcinoma due to non-alcoholic fatty liver disease. Expert Rev. Gastroenterol. Hepatol..

[6] Wong S.-W., Ting Y.-W., Chan W.-K. (2018). Epidemiology of non-alcoholic fatty liver disease-related hepatocellular carcinoma and its implications. JGH Open Open Access J. Gastroenterol. Hepatol..

[7] Mittal S., El-Serag H.B. (2013). Epidemiology of hepatocellular carcinoma: Consider the population. J. Clin. Gastroenterol..

[8] Simon J., Nuñez-García M., Fernández-Tussy P., Barbier-Torres L., Fernández-Ramos D., Gómez-Santos B., Buqué X., Lopitz-Otsoa F., Goikoetxea-Usandizaga N., Serrano-Macia M. (2020). Targeting Hepatic Glutaminase 1 Ameliorates Non-alcoholic Steatohepatitis by Restoring Very-Low-Density Lipoprotein Triglyceride Assembly. Cell Metab..

[9] Fernando D.H., Forbes J.M., Angus P.W., Herath C.B. (2019). Development and Progression of Non-Alcoholic Fatty Liver Disease: The Role of Advanced Glycation End Products. Int. J. Mol. Sci..

[10] Friedman S.L. (2007). Reversibility of hepatic fibrosis and cirrhosis—Is it all hype?. Nat. Clin. Pract. Gastroenterol. Hepatol..

[11] Canbay A., Friedman S., Gores G.J. (2004). Apoptosis: The Nexus of Liver Injury and Fibrosis. Hepatology.

[12] Canbay A., Feldstein A.E., Higuchi H., Werneburg N., Grambihler A., Bronk S.F., Gores G.J. (2003). Kupffer Cell Engulfment of Apoptotic Bodies Stimulates Death Ligand and Cytokine Expression. Hepatology.

[13] Mederacke I., Hsu C.C., Troeger J.S., Huebener P., Mu X., Dapito D.H., Pradere J., Schwabe R.F. (2013). Fate-tracing reveals hepatic stellate cells as dominant contributors to liver fibrosis independent of its etiology. Nat. Commun..

[14] Hallsworth K., Adams L.A. (2019). Lifestyle modification in NAFLD/NASH: Facts and figures. JHEP Rep. Innov. Hepatol..

[15] Paglialunga S., Dehn C.A. (2016). Clinical assessment of hepatic de novo lipogenesis in non-alcoholic fatty liver disease. Lipids Health Dis..

[16] Chooi Y.C., Ding C., Magkos F. (2019). The epidemiology of obesity. Metabolism.

[17] Greger J.L., Baligar P., Abernathy R.P., Bennett O.A., Peterson T. (1978). Calcium, magnesium, phosphorus, copper, and manganese balance in adolescent females. Am. J. Clin. Nutr..

[18] Ervin R.B., Wang C.-Y., Wright J.D., Kennedy-Stephenson J. (2004). Dietary intake of selected minerals for the United States population: 1999–2000. Adv. Data.

[19] Aikawa J.K. (1981). Magnesium: Its Biologic Significance.

[20] Swaminathan R. (2003). Magnesium metabolism and its disorders. Clin. Biochem. Rev..

[21] Liu M., Dudley S.C. (2020). Magnesium, Oxidative Stress, Inflammation, and Cardiovascular Disease. Antioxidants.

[22] Barbagallo M., Dominguez L.J., Galioto A., Ferlisi A., Cani C., Malfa L., Pineo A., Busardo’ A., Paolisso G. (2003). Role of magnesium in insulin action, diabetes and cardio-metabolic syndrome X. Mol. Asp. Med..

[23] Barbagallo M., Di Bella G., Brucato V., D’Angelo D., Damiani P., Monteverde A., Belvedere M., Dominguez L.J. (2014). Serum ionized magnesium in diabetic older persons. Metabolism.

[24] Rosique-Esteban N., Guasch-Ferré M., Hernández-Alonso P., Salas-Salvadó J. (2018). Dietary Magnesium and Cardiovascular Disease: A Review with Emphasis in Epidemiological Studies. Nutrients.

[25] Wu L., Zhu X., Fan L., Kabagambe E.K., Song Y., Tao M., Zhong X., Hou L., Shrubsole M.J., Liu J. (2017). Magnesium intake and mortality due to liver diseases: Results from the Third National Health and Nutrition Examination Survey Cohort. Sci. Rep..

[26] Liu M., Yang H., Mao Y. (2019). Magnesium and liver disease. Ann. Transl. Med..

[27] Rayssiguier Y., Chevalier F., Bonnet M., Kopp J., Durlach J. (1985). Influence of magnesium deficiency on liver collagen after carbon tetrachloride or ethanol administration to rats. J. Nutr..

[28] Panov A., Scarpa A. (1996). Mg^2+^ control of respiration in isolated rat liver mitochondria. Biochemistry.

[29] Konno Y., Ohno S., Akita Y., Kawasaki H., Suzuki K. (1989). Enzymatic properties of a novel phorbol ester receptor/protein kinase, nPKC. J. Biochem..

[30] Malpuech-Brugère C., Nowacki W., Daveau M., Gueux E., Linard C., Rock E., Lebreton J., Mazur A., Rayssiguier Y. (2000). Inflammatory response following acute magnesium deficiency in the rat. Biochim. Biophys. Acta.

[31] Paik Y.H., Yoon Y.J., Lee H.C., Jung M.K., Kang S.H., Chung S.I., Kim J.K., Cho J.Y., Lee K.S., Han K.H. (2011). Antifibrotic effects of magnesium lithospermate B on hepatic stellate cells and thioacetamide-induced cirrhotic rats. Exp. Mol. Med..

[32] Frick D.N., Banik S., Rypma R.S. (2007). Role of divalent metal cations in ATP hydrolysis catalyzed by the hepatitis C virus NS3 helicase: Magnesium provides a bridge for ATP to fuel unwinding. J. Mol. Biol..

[33] Blaszczyk U., Duda-Chodak A. (2013). Magnesium: Its role in nutrition and carcinogenesis. Rocz. Panstw. Zakl. Hig..

[34] Liu Y., Li X., Zou Q., Liu L., Zhu X., Jia Q., Wang L., Yan R. (2017). Inhibitory effect of magnesium cantharidate on human hepatoma SMMC-7721 cell proliferation by blocking MAPK signaling pathway. Chin. J. Cell. Mol. Immunol..

[35] Adachi M., Brenner D.A. (2005). Clinical syndromes of alcoholic liver disease. Dig. Dis..

[36] Adachi M., Ishii H. (2002). Role of mitochondria in alcoholic liver injury. Free Radic. Biol. Med..

[37] Weiskirchen R., Tacke F. (2014). Cellular and molecular functions of hepatic stellate cells in inflammatory responses and liver immunology. Hepatobiliary Surg. Nutr..

[38] Poikolainen K., Alho H. (2008). Magnesium treatment in alcoholics: A randomized clinical trial. Subst. Abus. Treat. Prev. Policy.

[39] Hruby A., Hu F.B. (2015). The Epidemiology of Obesity: A Big Picture. Pharmacoeconomics.

[40] Portillo-Sanchez P., Bril F., Maximos M., Lomonaco R., Biernacki D., Orsak B., Subbarayan S., Webb A., Hecht J., Cusi K. (2015). High Prevalence of Nonalcoholic Fatty Liver Disease in Patients With Type 2 Diabetes Mellitus and Normal Plasma Aminotransferase Levels. J. Clin. Endocrinol. Metab..

[41] Shamnani G., Rukadikar C., Gupta V., Singh S., Tiwari S., Bhartiy S., Sharma P. (2018). Serum magnesium in relation with obesity. Natl. J. Physiol. Pharm. Pharmacol..

[42] Castellanos-Gutiérrez A., Sánchez-Pimienta T.G., Carriquiry A., da Costa T.H.M., Ariza A.C. (2018). Higher dietary magnesium intake is associated with lower body mass index, waist circumference and serum glucose in Mexican adults. Nutr. J..

[43] Rodríguez-Moran M., Guerrero-Romero F. (2014). Oral magnesium supplementation improves the metabolic profile of metabolically obese, normal-weight individuals: A randomized double-blind placebo-controlled trial. Arch. Med. Res..

[44] Nielsen F.H. (2010). Magnesium, inflammation, and obesity in chronic disease. Nutr. Rev..

[45] Kurstjens S., van Diepen J.A., Overmars-Bos C., Alkema W., Bindels R.J.M., Ashcroft F.M., Tack C.J.J., Hoenderop J.G.J., de Baaij J.H.F. (2018). Magnesium deficiency prevents high-fat-diet-induced obesity in mice. Diabetologia.

[46] de Leeuw I.H., van Gaal L., Vanroelen W. (1987). Magnesium and obesity: Effects of treatment on magnesium and other parameters. Magnesium.

[47] Simental-Mendía L.E., Simental-Mendía M., Sahebkar A., Rodríguez-Morán M., Guerrero-Romero F. (2017). Effect of magnesium supplementation on lipid profile: A systematic review and meta-analysis of randomized controlled trials. Eur. J. Clin. Pharmacol..

[48] Mohamed-Ali V., Pinkney J.H., Coppack S.W. (1998). Adipose tissue as an endocrine and paracrine organ. Int. J. Obes..

[49] Devaux S., Adrian M., Laurant P., Berthelot A., Quignard-Boulangé A. (2016). Dietary magnesium intake alters age-related changes in rat adipose tissue cellularity. Magnes. Res..

[50] Antonopoulos A.S., Tousoulis D. (2017). The molecular mechanisms of obesity paradox. Cardiovasc. Res..

[51] Khan M.A.B., Hashim M.J., King J.K., Govender R.D., Mustafa H., Al Kaabi J. (2020). Epidemiology of Type 2 Diabetes—Global Burden of Disease and Forecasted Trends. J. Epidemiol. Glob. Health.

[52] Zheng Y., Ley S.H., Hu F.B. (2018). Global aetiology and epidemiology of type 2 diabetes mellitus and its complications. Nat. Rev. Endocrinol..

[53] Walti M.K., Zimmermann M.B., Walczyk T., Spinas G.A., Hurrell R.F. (2003). Measurement of magnesium absorption and retention in type 2 diabetic patients with the use of stable isotopes. Am. J. Clin. Nutr..

[54] McNair P., Christensen M.S., Christiansen C., Madsbad S., Transbøl I. (1982). Renal hypomagnesaemia in human diabetes mellitus: Its relation to glucose homeostasis. Eur. J. Clin. Investig..

[55] Mather H.M., Levin G.E. (1979). Magnesium status in diabetes. Lancet.

[56] Gommers L.M.M., Hoenderop J.G.J., Bindels R.J.M., de Baaij J.H.F. (2016). Hypomagnesemia in Type 2 Diabetes: A Vicious Circle?. Diabetes.

[57] Tin A., Grams M.E., Maruthur N.M., Astor B.C., Couper D., Mosley T.H., Selvin E., Coresh J., Kao W.H.L. (2015). Results from the Atherosclerosis Risk in Communities study suggest that low serum magnesium is associated with incident kidney disease. Kidney Int..

[58] Gutierrez-Rodelo C., Roura-Guiberna A., Olivares-Reyes J.A. (2017). Molecular Mechanisms of Insulin Resistance: An Update. Gac. Med. Mex..

[59] Kostov K. (2019). Effects of Magnesium Deficiency on Mechanisms of Insulin Resistance in Type 2 Diabetes: Focusing on the Processes of Insulin Secretion and Signaling. Int. J. Mol. Sci..

[60] Barbagallo M., Dominguez L.J. (2010). Magnesium and aging. Curr. Pharm. Des..

[61] Chen L., Chen R., Wang H., Liang F. (2015). Mechanisms Linking Inflammation to Insulin Resistance. Int. J. Endocrinol..

[62] Kandeel F.R., Balon E., Scott S., Nadler J.L. (1996). Magnesium deficiency and glucose metabolism in rat adipocytes. Metabolism.

[63] Verma H., Garg R. (2017). Effect of magnesium supplementation on type 2 diabetes associated cardiovascular risk factors: A systematic review and meta-analysis. J. Hum. Nutr. Diet. Off. J. Br. Diet. Assoc..

[64] Mills K.T., Stefanescu A., He J. (2020). The global epidemiology of hypertension. Nat. Rev. Nephrol..

[65] Saklayen M.G. (2018). The Global Epidemic of the Metabolic Syndrome. Curr. Hypertens. Rep..

[66] Singh S., Shankar R., Singh G.P. (2017). Prevalence and Associated Risk Factors of Hypertension: A Cross-Sectional Study in Urban Varanasi. Int. J. Hypertens..

[67] Ruan Y., Guo Y., Zheng Y., Huang Z., Sun S., Kowal P., Shi Y., Wu F. (2018). Cardiovascular disease (CVD) and associated risk factors among older adults in six low-and middle-income countries: Results from SAGE Wave 1. BMC Public Health.

[68] Benjamin E.J., Virani S.S., Callaway C.W., Chamberlain A.M., Chang A.R., Cheng S., Chiuve S.E., Cushman M., Delling F.N., Deo R. (2018). Heart Disease and Stroke Statistics-2018 Update: A Report From the American Heart Association. Circulation.

[69] Hinton W., McGovern A., Coyle R., Han T.S., Sharma P., Correa A., Ferreira F., de Lusignan S. (2018). Incidence and prevalence of cardiovascular disease in English primary care: A cross-sectional and follow-up study of the Royal College of General Practitioners (RCGP) Research and Surveillance Centre (RSC). BMJ Open.

[70] Dibaba D.T., Xun P., Song Y., Rosanoff A., Shechter M., He K. (2017). The effect of magnesium supplementation on blood pressure in individuals with insulin resistance, prediabetes, or noncommunicable chronic diseases: A meta-analysis of randomized controlled trials. Am. J. Clin. Nutr..

[71] Kawano Y., Matsuoka H., Takishita S., Omae T. (1998). Effects of magnesium supplementation in hypertensive patients: Assessment by office, home, and ambulatory blood pressures. Hypertension.

[72] Nguyen H., Odelola O.A., Rangaswami J., Amanullah A. (2013). A review of nutritional factors in hypertension management. Int. J. Hypertens..

[73] Tangvoraphonkchai K., Davenport A. (2018). Magnesium and Cardiovascular Disease. Adv. Chronic Kidney Dis..

[74] Efstratiadis G., Sarigianni M., Gougourelas I. (2006). Hypomagnesemia and cardiovascular system. Hippokratia.

[75] Resnick L.M., Militianu D., Cunnings A.J., Pipe J.G., Evelhoch J.L., Soulen R.L. (1997). Direct magnetic resonance determination of aortic distensibility in essential hypertension: Relation to age, abdominal visceral fat, and in situ intracellular free magnesium. Hypertension.

[76] Del Gobbo L.C., Song Y., Poirier P., Dewailly E., Elin R.J., Egeland G.M. (2012). Low serum magnesium concentrations are associated with a high prevalence of premature ventricular complexes in obese adults with type 2 diabetes. Cardiovasc. Diabetol..

[77] DiNicolantonio J.J., Liu J., O’Keefe J.H. (2018). Magnesium for the prevention and treatment of cardiovascular disease. Open Heart.

[78] Barbagallo M., Gupta R.K., Resnick L.M. (1996). Cellular ions in NIDDM: Relation of calcium to hyperglycemia and cardiac mass. Diabetes Care.

[79] Houston M. (2011). The role of magnesium in hypertension and cardiovascular disease. J. Clin. Hypertens..

[80] Rayssiguier Y., Gueux E., Bussière L., Durlach J., Mazur A. (1993). Dietary magnesium affects susceptibility of lipoproteins and tissues to peroxidation in rats. J. Am. Coll. Nutr..

[81] Berra-Romani R., Guzmán-Silva A., Vargaz-Guadarrama A., Flores-Alonso J.C., Alonso-Romero J., Treviño S., Sánchez-Gómez J., Coyotl-Santiago N., García-Carrasco M., Moccia F. (2019). Type 2 Diabetes Alters Intracellular Ca(2+) Handling in Native Endothelium of Excised Rat Aorta. Int. J. Mol. Sci..

[82] Gröber U., Schmidt J., Kisters K. (2015). Magnesium in Prevention and Therapy. Nutrients.

[83] Jahnen-Dechent W., Ketteler M. (2012). Magnesium basics. Clin. Kidney J..

[84] Kolisek M., Zsurka G., Samaj J., Weghuber J., Schweyen R.J., Schweigel M. (2003). Mrs2p is an essential component of the major electrophoretic Mg2+ influx system in mitochondria. EMBO J..

[85] Goytain A., Quamme G.A. (2005). Identification and characterization of a novel mammalian Mg^2+^ transporter with channel-like properties. BMC Genom..

[86] Schlingmann K.P., Waldegger S., Konrad M., Chubanov V., Gudermann T. (2007). TRPM6 and TRPM7-Gatekeepers of human magnesium metabolism. Biochim. Biophys. Acta Mol. Basis Dis..

[87] Chen Y.S., Kozlov G., Fakih R., Funato Y., Miki H., Gehring K. (2018). The cyclic nucleotide-binding homology domain of the integral membrane protein CNNM mediates dimerization and is required for Mg(2+) efflux activity. J. Biol. Chem..

[88] Matsuda-Lennikov M., Biancalana M., Zou J., Ravell J.C., Zheng L., Kanellopoulou C., Jiang P., Notarangelo G., Jing H., Masutani E. (2019). Magnesium transporter 1 (MAGT1) deficiency causes selective defects in N-linked glycosylation and expression of immune-response genes. J. Biol. Chem..

[89] Kuramoto T., Kuwamura M., Tokuda S., Izawa T., Nakane Y., Kitada K., Akao M., Guénet J.-L., Serikawa T. (2011). A mutation in the gene encoding mitochondrial Mg^2^+ channel MRS2 results in demyelination in the rat. PLoS Genet..

[90] Fleig A., Schweigel-Röntgen M., Kolisek M. (2013). Solute carrier family SLC41: What do we really know about it?. Wiley Interdiscip. Rev. Membr. Transp. Signal..

